# A case of discrepancy between three ERA tests in a woman with repeated implantation failure complicated by chronic endometritis

**DOI:** 10.1186/s12884-022-05241-6

**Published:** 2022-12-01

**Authors:** Kuniaki Ota, Toshifumi Takahashi, Junichiro Mitsui, Kishio Kuroda, Kenichiro Hiraoka, Kiyotaka Kawai

**Affiliations:** 1 Fukushima Medical Center for Children and Women, Fukushima Medical University, 1 Hikarigaoka, Fukushima, 960-1295 Japan; 2 Kameda IVF Clinic Makuhari, Makuhari, 261-8501 Japan; 3 Department of Pathology, Nagasaki University, Nagasaki, 852-8501 Japan

**Keywords:** Endometrial receptivity array, Chronic endometritis, CD138, Plasma cell

## Abstract

**Background:**

Endometrial receptivity array (ERA) is used to determine the timing of embryo transfer (ET) synchronized with the window of implantation (WOI). The effectiveness and evaluation of ERAs in women with recurrent implantation failure remain controversial. We report the case of a patient with recurrent implantation failure that raises the issue of reproducibility of ERA tests.

**Case report:**

A 36-year-old Japanese woman with secondary infertility who had previously given birth failed to conceive after three frozen-thawed embryo transfer (FET) cycles. An ERA test was conducted to confirm the WOI. The first ERA test was performed 125 h after progesterone exposure. The laboratory reported that the endometrium was in a non-receptive (post-receptive) phase, and recommended retesting 101 h after progesterone exposure. A simultaneous chronic endometritis (CE) test showed a score of 3. After the antibiotics administration to treat CE, the second ERA test was performed after 101 h of progesterone exposure. The laboratory reported that the endometrium had not reached the WOI and estimated the WOI to be 113 ± 3 h after progesterone exposure. The third ERA test was performed 113 h after progesterone exposure. The laboratory reported that the endometrium was in a non-receptive (pre-receptive) phase and estimated the WOI to be 137 ± 3 h after progesterone exposure. A CE test performed at the same time as the second and third ERA tests showed a score of 1 for the collected endometrium. According to the third ERA test results, the vitrified-warmed blastocyst was transferred at 137 h of progesterone exposure. Pregnancy was achieved and the patient had an uncomplicated vaginal delivery at 39 weeks. One year later, another pregnancy was achieved after FET at 137 h of progesterone exposure, and the patient delivered at 33 weeks due to an unexpected membrane rupture.

**Conclusion:**

Because the results of the ERA test may vary in the presence of CE, CE should be diagnosed simultaneously with or before conducting ERA tests. If CE is diagnosed, ERA testing should be performed after treatment with antimicrobials or other drugs.

## Background

Endometrial receptivity is limited to 4–5 days during blastocyst implantation. The implantation period of the endometrium is called the window of implantation (WOI), during which the secretion of various cytokines in response to ovarian steroid hormones is regulated [1, 2].

In assisted reproduction technology (ART) treatment of the frozen-thawed embryo transfer (FET) cycle, endometrial preparation is pharmacologically mimicked by hormone replacement treatment with estrogen and progesterone and monitored for the WOI-assessed endometrial thickness measured by ultrasonography and blood hormone levels [3, 4]. These methods do not assess endometrial receptivity for objectivity and precision; therefore, a better way of objectively and reproducibly assessing endometrial receptivity is required.

Endometrial receptivity-associated genes have been previously investigated in humans [5, 6]. In 2011, Díaz-Gimeno et al. identified the transcriptome of 238 genes expressed at different stages of the endometrial cycle [6]. They named the results of this test endometrial receptivity array (ERA) and advocated personalizing ET (pET) according to the estimated individual WOI [7]. The pET, according to the ERA test, has been implemented in clinical practice [8]; however, the effectiveness and evaluation of pET in women with recurrent implantation failure (RIF) remains controversial [9–12].

Evidence has accumulated regarding the adverse effects of chronic endometritis (CE) and ART treatment. CE is characterized by persistent inflammation of the endometrium. CE is histologically diagnosed based on the presence of an excessive number of CD138-positive plasma cells infiltrating endometrial biopsies [13, 14]. CE significantly impairs the implantation process by affecting endometrial decidualization and results in poor pregnancy outcomes in women with RIF [15]. According to a recent report, endometrium with CE may change individual WOIs, and the results of ERA tests do not match the exact timing of pET in the presence of CE [16]. However, whether CE has an impact on ERA testing was not determined because the sample collection times for CE and ERA testing were different.

In our case, a woman with secondary infertility who had previously given birth failed to conceive after three FET cycles, and an ERA test was conducted to confirm the WOI. In simultaneous ERA and CE tests, endometrial samples with CE did not yield the recommended time of implantation in the ERA test. The ERA test was repeated after CE treatment and ET was performed based on the results of the third ERA test, resulting in pregnancy.

## Case presentation

A 36-year-old Japanese woman (gravida 1, para 1) visited our clinic because of secondary infertility. Her first pregnancy at 31 years of age was natural and she delivered a 3320 g infant by vaginal delivery at 39 weeks and 5 days of gestation without perinatal complications. The patient had regular menstrual cycles for 30 days. Her body mass index (BMI) was 18.6 (height 159.5 cm, weight 47.2 kg). Medical and family history were unremarkable. An internal examination revealed no abnormalities in the uterus or ovaries. Ultrasound examination revealed a polycystic pattern in the right ovary; however, the patient was not diagnosed with polycystic ovarian syndrome. Hormone levels of anti-Müllerian hormone (AMH), cycle day 3 (CD 3) of estradiol (E2), follicle-stimulating hormone (FSH), and luteinizing hormone (LH) were 7.44 ng/mL, 23.0 pg/mL, 7.3 mU/mL, and 7.4 mU/mL, respectively. Hysterosalpingography showed bilateral fallopian tube passage. The husband’s semen analysis revealed no abnormalities in semen volume, sperm count, or sperm motility according to the WHO 2010 criteria.

After five cycles of timed intercourse followed by five cycles of intrauterine insemination with the husband’s semen, no pregnancy was established. Therefore, the patient underwent in vitro fertilization (IVF). Controlled ovarian stimulation was performed using a gonadotropin-releasing hormone (GnRH) antagonist protocol. Purified urinary FSH (150 IU, Gonapure, ASKA Pharmaceutical, Tokyo, Japan) was administered every alternate day, and clomiphene citrate (50 mg/day, Clomid, Shionogi Co. Ltd., Osaka, Japan) for 5 days was started on CD 3. On CD 10, human menopausal gonadotropin (225 IU, Ferring Pharma, Tokyo, Japan) with GnRH antagonist (0.25 mg, Ganirest subcutaneous syringes, MSD, Tokyo, Japan) were administrated. On CD 12, transvaginal ultrasound showed 14 follicles larger than 16 mm, and the serum E2 level was 1332 pg/mL. Final oocyte maturation was triggered by subcutaneous injection of recombinant human chorionic gonadotropin (rhCG) (0.25 μg, Ovidrel, Serono Inc., Tokyo, Japan) and nasal spray of a GnRH agonist (600 μg, Buserecure, Fuji Pharma, Tokyo, Japan). Oocyte retrieval was performed 35 h after the trigger under general anesthesia. A total of 15 cumulus-oocyte complexes were retrieved, and 12 oocytes reached the MII stage. Conventional IVF and intracytoplasmic sperm injection (ICSI) were performed according to semen parameters. All embryos were cultured in ONESTEP Medium (NAKA Medical Inc., Tokyo, Japan) under 6% O_2_, 5% CO_2_, and 90% N_2_ gas. Cleavage-stage embryo quality was evaluated on day 3 according to Veeck’s criteria [17] and blastocysts were evaluated according to Gardner’s classification [18]. One cleavage embryo (10 cells, G2) and seven blastocysts (three blastocysts for 4AA, one blastocyst for 4AB, and two blastocysts for 4BB) were cryopreserved by vitrification using the Cryotop carrier system (Kitazato Biopharma Co., Tokyo, Japan) according to the manufacturer’s instructions.

FET was scheduled, and endometrial preparation was performed using a hormone replacement cycle (HRC) or modified natural cycle. In the HRC, transdermal estradiol (0.72 mg, Estrana TAPE, Hisamitsu Pharmaceutical, Tokyo, Japan) was initiated on CD 3. Progesterone treatment with vaginal progestin tablets (300 mg/day, LUTINUS Vaginal Tablet, Ferring Pharmaceuticals Co., Ltd., Tokyo, Japan) and oral dydrogesterone tablets (30 mg/day, Duphaston, Mylan EPD, Tokyo, Japan) was initiated at an endometrial thickness of 8 mm. FET was scheduled 3–5 days after the start of progesterone treatment. In the modified natural cycle, rhCG (0.25 μg, Ovidrel, Serono Inc., Tokyo, Japan) was injected when the endometrial thickness and follicle diameter reached more than 8 and 16 mm, respectively. Vitrified-warmed ET was scheduled 4–6 days after rhCG administration, according to the embryo stage.

In the first cycle of FET, one blastocyst-graded 4BB was transferred under the HRC. In the second cycle of FET, one early cleaved embryo, graded G2, was transferred under a modified natural cycle. In the third cycle of FET, one blastocyst-graded 4AB was transferred under a modified natural cycle. All embryos used in these three embryo transfers were of conventional IVF origin. However, the three FET cycles did not result in pregnancy.

After three cycles of FET failure, the patient decided to undergo an ERA test (IGENOMIX, Valencia, Spain). Endometrial preparation for the ERA test was performed using the standard HRC described above. The initial day of progesterone administration was set as 0 h of progesterone exposure. Endometrial sampling was performed after 125 h of progesterone exposure using a Pipelle endometrial sampler (Laboratoire CCD, Paris, France). Specimens were processed and shipped according to the manufacturer’s instructions. The ERA results were tabulated as reported by IGENOMIX and classified as receptive or non-receptive. Non-receptive results were considered either pre-or post-receptive, and details of endometrial adjustment recommendations or rebiopsy were documented. In addition, the presence or absence of CE in the biopsied endometrium was examined. The diagnostic criteria for CE were based on a previous study [19]. Accordingly, CE was defined as the presence of one or more plasma cells/high-power field (hpf) (× 40) in CD138 immunostaining and classified as score 1 for 1–5 cells/hpf, score 2 for 6–20 cells/hpf, and score 3 for more than 20 cells/hpf.

**Fig. 1:**
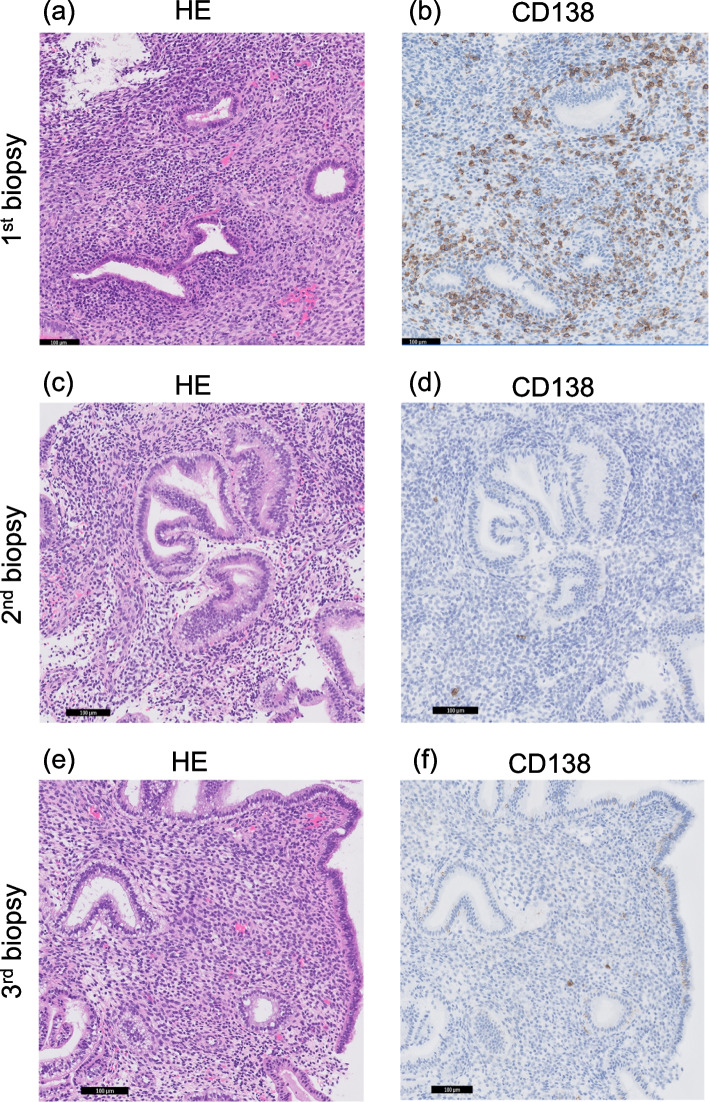
Diagnosis of chronic endometritis based on histopathology findings of endometrial tissue collected at the same time as the three ERA tests. Endometrial tissue was analyzed by hematoxylin and eosin (HE, left side), and by immunohistochemistry for CD138 staining (right side) (Magnification, × 400). **a**, **b** Images obtained from the first biopsy, **c**, **d** second biopsy and **e**, **f** third biopsy. ERA: endometrial receptivity array. These photographs were taken using a digital camera (Dalsa Piranha high-speed line scan camera, Teledyne DALSA, Ontario, Canada) with an Olympus microscope (Olympus, Tokyo, Japan) and a 20× objective lens. The images were edited using image management software (Philips Image Management System, Royal Philips, Amsterdam, The Netherlands). The black bar in each photo indicates 100 μm

The first ERA test was performed 125 h after progesterone exposure. The laboratory reported that the endometrium was in a non-receptive (post-receptive) phase, and recommended retesting 101 h after progesterone exposure. A simultaneous CE test showed a score of 3 (Fig. 1a and b). She was administered antibiotics for 2 weeks with levofloxacin 0.5 g/day and metronidazole (1 g/day) to treat CE. Subsequently, the second ERA test was performed after 101 h of progesterone exposure. The laboratory reported that the endometrium had not reached the WOI and estimated the WOI to be 113 ± 3 h after progesterone exposure. We questioned the results of the estimated WOI and completed the third ERA test after obtaining informed consent from the patient. The third ERA test was performed 113 h after progesterone exposure. The laboratory reported that the endometrium was in a non-receptive (pre-receptive) phase and estimated the WOI to be 137 ± 3 h after progesterone exposure. A CE test performed at the same time as the second and third ERA tests showed a score of 1 for the collected endometrium (Fig. 1c, d, e, and f).

**Fig. 2:**
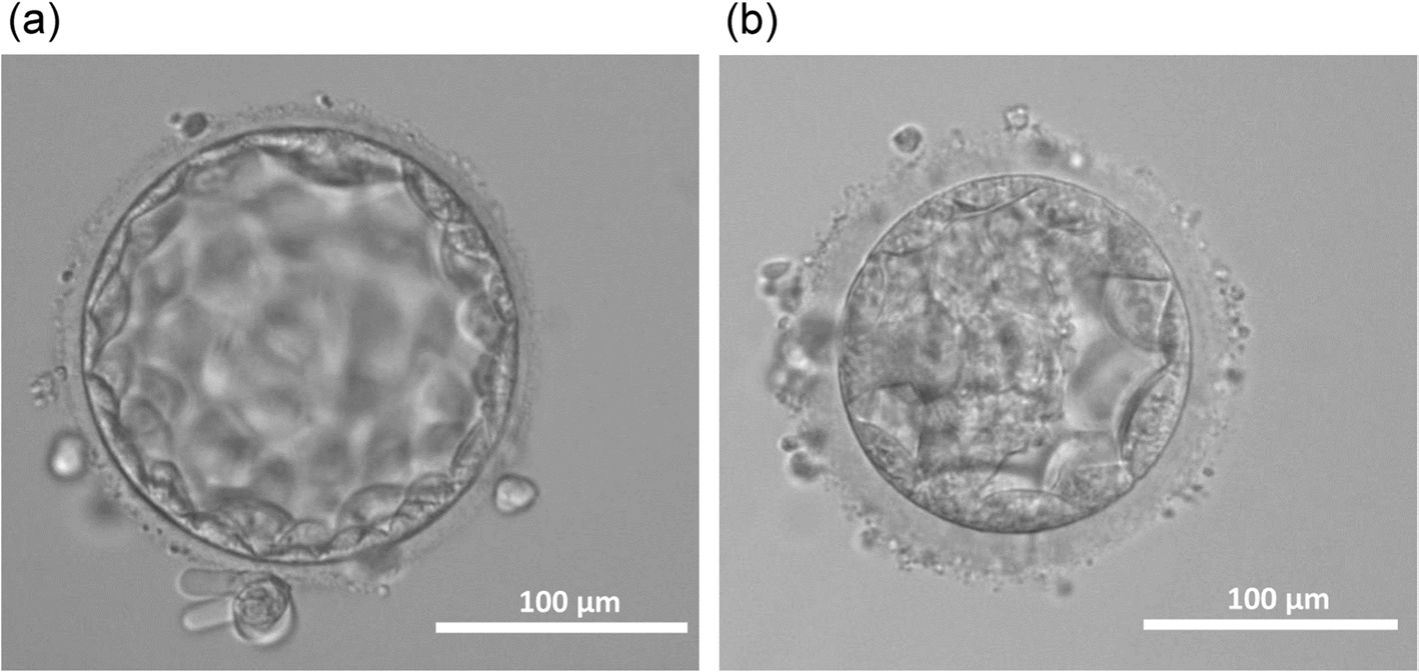
Photograph of blastocysts with successful pregnancies. **a** In the window of implantation as 113 ± 3 h after progesterone exposure, a vitrified-warmed blastocyst graded to 4 AA was transferred, resulting in a successful pregnancy and delivery. **b** One year after the first delivery, another vitrified-warmed blastocyst graded to 4 AA was transferred with the same window of implantation, resulting in a second successful pregnancy and delivery. Photographs of the two blastocysts were taken using a digital camera (BU130, Toshiba Teli, Tokyo, Japan) with a Nikon microscope (Nikon, Tokyo, Japan) and 10× objective lens. The captured images were edited using image management software (Phototune, CCM-BIS viewer soft, ASTEC, Fukuoka, Japan). The white bar in each photo indicates 100 μm

**Fig. 3:**
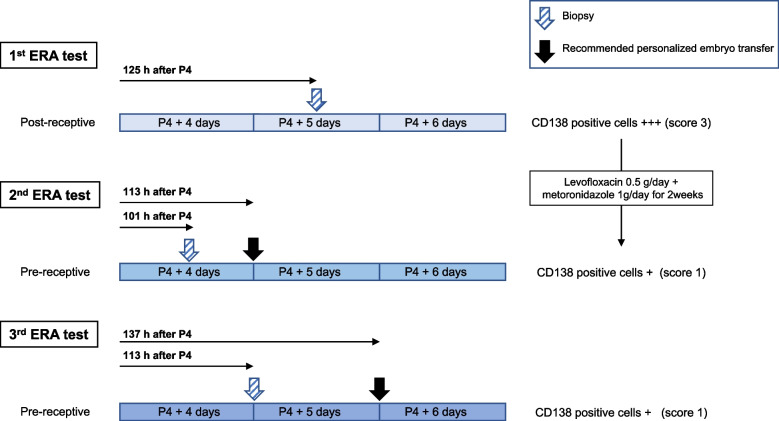
Scheme of the timing of endometrial sampling and the recommended window of implantation by the three ERA tests. The process of endometrial profiling based on endometrial receptivity array test results and the diagnosis of chronic endometritis with endometrial biopsy. ERA: endometrial receptivity array

According to the third ERA test results, the vitrified-warmed blastocyst graded 4AA (Fig. 2a), which was derived from ICSI, was transferred at 137 h of progesterone exposure, which corresponded to ET 12 h later than the normal HRC. Pregnancy was achieved in this ET cycle, and the patient had an uncomplicated vaginal delivery of a male neonate weighing 2970 g at 39 weeks and 5 days. One year later, another FET of one 4AA blastocyst (Fig. 2b) was performed in the HRC, and pregnancy was achieved after 137 h of progesterone exposure. She delivered a viable 2168 g female neonate at 33 weeks and 2 days after an unexpected rupture of the membrane occurred at 33 weeks and 1 day. The timing of endometrial sampling and the recommended WOI by the three ERA tests are shown in Fig. 3.

## Discussion and conclusions

In the present case, an ERA test was conducted to confirm the WOI in a woman with secondary infertility who previously failed to conceive after three FETs. The results of the ERA test showed non-receptive (post-receptive), however, the staining results of CD138-positive cells in the endometrium performed at the same time showed a complication of CE. The ERA test performed after CE treatment resulted in the pre-receptive phase. As CE is likely to affect the results of the ERA test, it is better to perform CE tests at the same time as ERA tests.

The ERA test estimates the WOI based on changes in the expression of implantation-related genes in the endometrium during the HRT cycle. Therefore, various factors affect the estimated WOI. Factors that have been reported to affect the results of the ERA test include the administration route of progesterone, cumulative dose of administered progesterone, levels of progesterone and its metabolites in the local endometrium, patient’s BMI, and presence of CE. Barrenetxea et al. examined the ERA test and route of progesterone administration [20]. They revealed that more patients were in the receptive phase after oral administration of micronized progesterone than after vaginal administration. They also reported that BMI and cumulative progesterone administration were independently associated factors in multivariate analysis of the receptive phase. Labarta et al. examined the relationship between the ERA test and serum and endometrial progesterone levels [21]. They reported that although the receptive phase in the ERA test was not related to serum progesterone levels, it was associated with endometrial progesterone and its metabolite 17α-hydroxyprogesterone levels. In the present case, the patient had a normal BMI that did not fluctuate during multiple ERA examinations. In addition, the patient received vaginal micronized progesterone during all ERA examinations. The effect of these factors has not been fully explained for the ERA test discrepancy in the present patient.

There are very few clinical studies on CE and ERA tests. Recently, Kuroda et al. reported that the percentage of patients in the receptive phase was significantly lower in patients with CE than in patients without CE or after CE treatment, which is consistent with our observations. The following reports provide evidence to explain the possibility of WOI misalignment on the ERA test in patients with CE. Di Pietro et al. examined gene expression in the implantation endometria of patients with and without CE. They reported that in patients with CE, endometrial expression of IGFBP1, BCL2, and BAX genes was upregulated, whereas that of IL11, CCL4, IGF1, and CASP8 was downregulated [22]. Wu et al. reported that endometrial stromal cells (ESCs) in patients with CE had lower secretion of prolactin and IGFBP1 in vitro than those without CE [15]. They further reported that estrogen- and progesterone-stimulated decidualization of ESCs was significantly impaired in patients with CE than in those without CE [15]. Taken together, these reports support the idea that CE may disturb endometrial gene expression and ovarian steroid-induced decidualization during the implantation period. However, the relationship between ERA test results and CE needs to be further examined.

The appropriateness of performing CE testing at the same time as the ERA test is discussed. Endometrial sampling, as a test for CE, is often performed during the proliferative phase of the same menstrual cycle as hysteroscopy. The number of CD138 positive cells changes during the menstrual cycle, decreasing during the luteal phase than during the proliferative phase [23]. However, it has not been reported whether estrogen and progesterone administration during the HRT cycle affects the CD138 positive cell count. In the present case, the endometrial sample was examined at a time corresponding to the luteal phase of the normal menstrual cycle, which may have underestimated the CD138 positive cell count. Further studies are needed to determine the timing of CE and ERA testing.

In the present case, good blastocysts failed to implant in the patient after FET. New treatments for RIF are emerging; however, many of them do not have established evidence. The clinical evaluation of ERA in patients with RIF and non-RIF has been controversial. pET determined by ERA has been reported to contribute to statistically significant improvements in pregnancy, implantation, and cumulative live birth rates in FET cycles [24, 25]. Patel et al. reported the results of a retrospective observational cross-sectional study of the efficacy of ERA in patients with RIF [24]. They performed ERA in 248 patients with RIF and reported that 82.3% of patients were receptive, 17.7% were non-receptive, 61.4% were pre-receptive and 38.6% were post-receptive. One hundred and seventy-five receptive patients underwent ET with no change in time after progesterone administration in FET cycles. The clinical pregnancy rates of receptive and non-receptive patients who underwent ET and pET were 49.7 and 45.7%, respectively. In a recent study, 458 women under 37 years of age who underwent blastocyst transfer were randomly assigned to one of three groups (pET by ERA, FET, and fresh ET) and the implantation rate in the first ET cycle was examined. The implantation rate with pET using ERA was significantly higher than that with FET and fresh ET [25].

The factors that influenced whether a pregnancy was achieved in this case involved the presence or absence of WOI shift and CE and the embryo’s developmental potential. An important factor affecting embryo development is the aneuploidy of the embryo, and preimplantation genetic testing for aneuploidy (PGT-A) is necessary to test the aneuploidy of embryos before transfer. However, in our country, the requirements for performing PGT-A are strict, and it is not easy to perform PGT-A on all embryos. Further research should be needed on the effectiveness of ERA test and CE treatment using euploid embryos.

However, studies have reported that pET based on the results of the ERA test did not significantly improve pregnancy outcomes [9, 12]. Using a retrospective cohort study, Tan et al. reported that both implantation and ongoing pregnancy rates in patients with a history of failed euploid FET with guided pET in the ERA test tended to be increased (73.7 vs. 54.2% and 63.2 vs. 41.7%, respectively) compared to patients who did not undergo the ERA test, although the differences were not statistically significant [9]. Bassil et al. retrospectively examined the pregnancy rates of 41 women who underwent pET with ERA and 503 women who underwent FET without ERA, and both groups’ pregnancy rates were comparable [12]. A recent review pointed out the limitations and problems of ERA testing [26]; studies on the usefulness of ERA should be continued.

We report the case of a patient with RIF that raises the issue of reproducibility of the ERA test. The diagnosis of CE should be made at the same time or before ERA testing. If CE is diagnosed, ERA testing should be performed after treatment with antimicrobials or other drugs.

## Data Availability

The data that support the findings of this study are available on request from the corresponding author. The data are not publicly available due to privacy or ethical restrictions.

## Abbreviations

ERA: Endometrial receptivity array
ET: Embryo transfer
WOI: Window of implantation
CE: Chronic endometritis
ART: Assisted reproduction technology
FET: Frozen-thawed embryo transfer
PET: Personalizing ET
RIF: Recurrent implantation failure
BMI: Body mass index
AMH: Anti-Müllerian hormone
CD: Cycle day
E2: Estradiol
FSH: Follicle-stimulating hormone
LH: Luteinizing hormone
IVF: In vitro fertilization
rhCG: Recombinant human chorionic gonadotropin
ICSI: Intracytoplasmic sperm injection
HRC: Hormone replacement cycle
PGT-A: Preimplantation genetic testing for aneuploidy
